# Size, not temperature, drives cyclopoid copepod predation of invasive mosquito larvae

**DOI:** 10.1371/journal.pone.0246178

**Published:** 2021-02-02

**Authors:** Marie C. Russell, Alima Qureshi, Christopher G. Wilson, Lauren J. Cator

**Affiliations:** Department of Life Sciences, Imperial College London, Ascot, United Kingdom; University of Connecticut, UNITED STATES

## Abstract

During range expansion, invasive species can experience new thermal regimes. Differences between the thermal performance of local and invasive species can alter species interactions, including predator-prey interactions. The Asian tiger mosquito, *Aedes albopictus*, is a known vector of several viral diseases of public health importance. It has successfully invaded many regions across the globe and currently threatens to invade regions of the UK where conditions would support seasonal activity. We assessed the functional response and predation efficiency (percentage of prey consumed) of the cyclopoid copepods *Macrocyclops albidus* and *Megacyclops viridis* from South East England, UK against newly-hatched French *Ae*. *albopictus* larvae across a relevant temperature range (15, 20, and 25°C). Predator-absent controls were included in all experiments to account for background prey mortality. We found that both *M*. *albidus* and *M*. *viridis* display type II functional response curves, and that both would therefore be suitable biocontrol agents in the event of an *Ae*. *albopictus* invasion in the UK. No significant effect of temperature on the predation interaction was detected by either type of analysis. However, the predation efficiency analysis did show differences due to predator species. The results suggest that *M*. *viridis* would be a superior predator against invasive *Ae*. *albopictus* larvae due to the larger size of this copepod species, relative to *M*. *albidus*. Our work highlights the importance of size relationships in predicting interactions between invading prey and local predators.

## Introduction

An invasion of the Asian tiger mosquito, *Aedes albopictus*, into the UK is a major public health concern. This species is not only an aggressive nuisance biter, but also a known vector of arboviruses such as dengue, chikungunya, yellow fever, and Zika [1]. The ability of *Ae*. *albopictus* mosquitoes to lay desiccation-resistant eggs has enabled their introduction into cities across the globe, often via used tire shipments [2–6]. According to the European Centre for Disease Prevention and Control, *Ae*. *albopictus* populations have already established throughout the vast majority of Italy and southern France [7]. Although this species has not yet established in the UK, it has been introduced in Kent, a coastal county in the southeast of the UK, where 37 *Ae*. *albopictus* eggs were found in September of 2016 [7, 8]. Owing to the warming climate, the UK is expected to become increasingly suitable for *Ae*. *albopictus* establishment; projections of climate and human population conditions into future decades suggest that if introduced, the vector species could establish throughout most of England and southern Wales during the 2060s [9]. Another recent model predicts that the UK will report *Ae*. *albopictus* presence by either 2050 or 2080, depending on the patterns of future greenhouse gas emissions [10].

Predation by copepods has been identified as a biological method for controlling *Ae*. *albopictus* in Europe following the success of past field trials [11]. Cyclopoid copepods from India, the US, Australia, Vietnam, and Italy, including copepod species from the genera *Macrocyclops* and *Mesocyclops*, have proven to be effective predators of local mosquito larvae [12–17]. In the UK, laboratory experiments using cyclopoid copepods from Northern Ireland against *Culex pipiens* mosquito larvae from Surrey, UK and *Ae*. *albopictus* larvae from Montpellier, France have supported the use of copepods as biocontrol agents [18, 19]. Previous work indicates that the attack rates of *Macrocyclops albidus* and *Megacyclops viridis* predators against *Cx*. *pipiens* prey tend to increase with temperature [19]. However, the use of UK copepods as predators of invasive *Ae*. *albopictus* larvae has not been thoroughly examined over the range of temperatures that the invasive larvae are predicted to experience. Due to the potentially negative impacts of exporting copepods to non-native regions for biocontrol purposes [20], it is important to investigate the performance of copepods local to the predicted sites of *Ae*. *albopictus* establishment: London and South East England [9].

Populations of *M*. *albidus* and *M*. *viridis* cyclopoid copepods from the benthos of the Cumbrian lakes in the UK have previously been studied at temperatures from 5 to 20°C [21]. Based on the dry body masses of adult specimens, males were consistently smaller than females, and *M*. *albidus* copepods were consistently smaller than *M*. *viridis* [21]. The general adult body length ranges for *M*. *albidus* and *M*. *viridis* are 1.3–2.5 mm [22] and 1.2–3 mm [23–25], respectively. These are small enough to enable the distribution of copepod cultures to mosquito larval habitats using “a simple backpack sprayer with a 5 mm hole in the nozzle,” as has been previously recommended [26]. The application of cyclopoid copepods to control mosquitoes has been described as efficient, safe, and economical [27]. Copepods reproduce sexually, and females that can produce new egg sacs every 3–6 days tend to predominate in mature populations [28]. Although adults have been observed consuming immatures of the nauplius stage, these instances of cannibalism are not common when other prey options are available [29]. Cyclopoid copepods are considered sit-and-wait ambush predators because of their attack behaviors, which have been described in six steps: encounter, aiming, stalking, attack, capture, and ingestion [30].

Functional response curves were originally developed to relate the number of prey attacked by an invertebrate predator to the prey density [31, 32]. Previous work has suggested that the best predators to use in an “inundative release” biocontrol program are those that have type II functional responses to prey density, so that the predators’ attack rates are high even at low prey densities [33]. Predators that display a type III functional response against an invasive prey species—such as signal crayfish (*Pacifastacus leniusculus*) against New Zealand mud snails (*Potamopyrgus antipodarum*)—may be able to limit the spread of the invader, but cannot prevent it from establishing [34]. Cyclopoid copepods from the UK have previously exhibited type II functional response curves when provided with *Ae*. *albopictus* prey at a single temperature setting [18]. A meta-analysis of fifty functional response curves of cyclopoid copepod predators showed a “monotonically increasing effect of temperature” on the attack rate parameter, which is used to measure foraging [35]. However, recent literature shows that the temperature dependence of attack rate is unimodal, or “hump-shaped,” especially when the temperature range exceeds thermal optima, and suggests that the impact of climate change on predation will depend on the thermal optima of consumers [36, 37].

In this study, we determined the type of functional response of *M*. *albidus* and *M*. *viridis* copepods from Surrey, UK against *Ae*. *albopictus* larvae from Montpellier, France at three different temperatures (15, 20, and 25°C) likely to be experienced by container-dwelling mosquito larvae in London and South East England. We also calculated predation efficiency, the percentage of larvae consumed, of both copepod species at a constant mosquito prey density under the same three temperatures.

## Materials and methods

### Collection of field temperature data

Previous work has suggested that the microclimates experienced by *Ae*. *albopictus* are not represented well by climate data reported from local weather stations [38]. Since used tire shipments are a common source of *Ae*. *albopictus* introductions [2–6], we measured the temperature of rainwater collected in used car tires that were divided between an urban London site and a suburban site (S1 Fig, S1 File). These temperature data provided rough guidelines for our choice of the three temperatures to be tested in our functional response and predation efficiency experiments. The minimum water temperature was 9°C, the 25^th^ percentile was 16.1°C, the median was 18.1°C, the 75^th^ percentile was 20°C, and the maximum was 25°C (S2 Fig). Thus, our choice of 15, 20, and 25°C as the three main temperatures of interest is representative of more than 75% of the tire water temperatures recorded in the field from May through September of 2018.

### Local copepod cultures

Adult gravid female copepods were collected in August of 2018 from the edge of Longside Lake in Egham, Surrey, UK (N 51° 24.298’, W 0° 32.599’) using a 53 μm sieve (Reefphyto Ltd, UK). Neither the collection of UK aquatic invertebrates, nor the use of these organisms in laboratory experiments, currently requires permission from an ethics committee; and access to Longside Lake was not restricted at the time of sampling. Separate cultures were started from each gravid female. The copepods were kept in 3 L containers of spring water (Highland Spring, UK) at a 12:12 light/dark cycle, and 20 ± 1°C, a temperature previously found to increase the portion of their life cycle spent in the reproductive phase [21]. *Chilomonas paramecium* and *Paramecium caudatum* (Sciento, UK) were provided *ad libitum* as food for the copepods [39]. The ciliates were cultured in 2 L flasks containing boiled wheat seeds; boiled wheat seeds were also added to the copepod containers [39]. Adult copepods were identified as *Macrocyclops albidus* (Jurine, 1820) and *Megacyclops viridis* (Jurine, 1820) by Dr. Maria Hołyńska from the Museum and Institute of Zoology in Warsaw, Poland. The copepod cultures were maintained continuously for approximately three months before the start of the experiments.

### Temperate *Ae*. *albopictus* colony care

A colony of *Ae*. *albopictus* mosquitoes (original collection Montpellier, France 2016 obtained through Infravec2) was maintained at 27 ± 1°C, 70% relative humidity, and a 12:12 light/dark cycle. The colony maintenance temperature of 27°C was chosen because it falls at the upper bound of the 95% confidence interval that was fitted around mean daily Montpellier temperatures from 1996–2005 in the summer months, and these temperatures are expected to increase in the coming decades [40]. Larvae were fed fish food (Cichlid Gold Hikari®, Japan), and adults were given 10% sucrose solution and horse blood (First Link Ltd, UK) administered through a membrane feeding system (Hemotek®, Blackburn, UK).

### Design of functional response experiments

**Fig 1:**
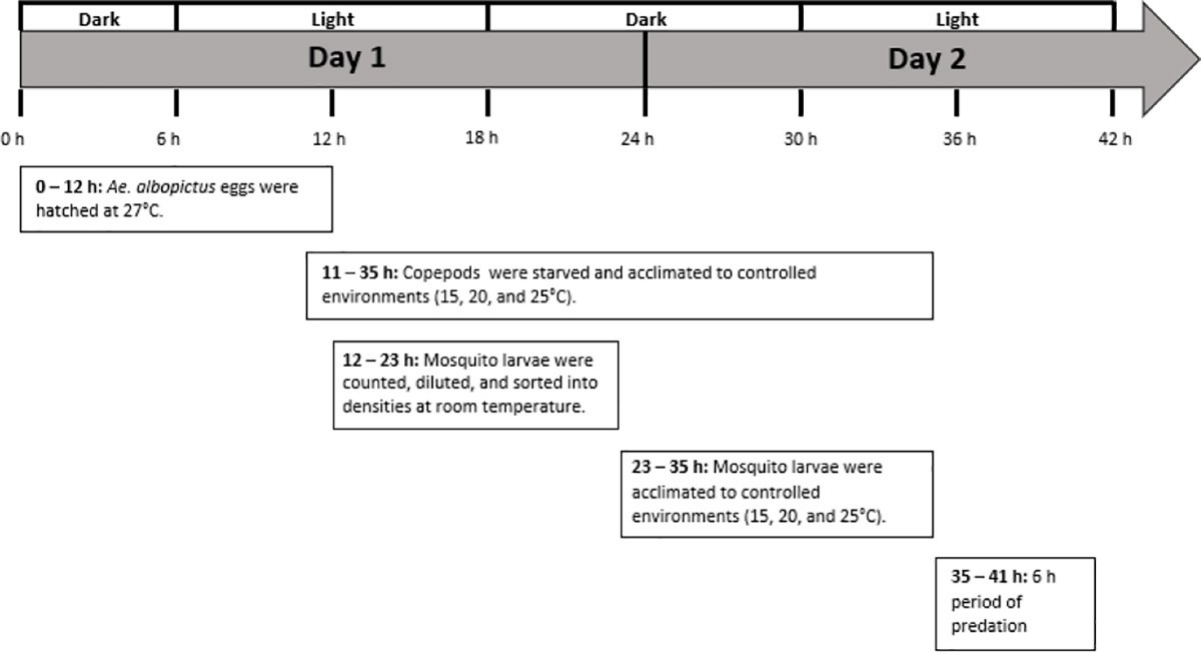
General schedule of both functional response and predation efficiency experiments.

Adult non-gravid female copepods, identified by larger relative size, were removed from their culture and each was placed in a Petri dish (diameter: 50 mm, height: 20.3 mm) holding 20 mL of spring water. The copepods were placed in three different controlled environments set to 15 ± 1, 20 ± 1, and 25 ± 1°C, all at a 12:12 light/dark cycle, to begin a 24 h starvation and temperature-acclimation period for the predators (Fig 1). The temperature and light cycle settings were maintained by two computer-programmed Panasonic incubators and one computer-programmed Weiss Technik: Fitotron® controlled environment room. Each combination of copepod species (*M*. *albidus* or *M*. *viridis*) and temperature had 28 copepods, each one held in its own Petri dish; there were 168 copepods in total (S3 Fig). Ten additional gravid females were randomly selected from each species of copepod and preserved in 80% ethanol for size measurements.

Groups of 87 newly-hatched *Ae*. *albopictus* larvae (S2 File) were each pipetted into a small plastic tub containing 150 mL of spring water to strongly dilute any residual food from the hatching media. Each tub of 87 larvae was then split into seven Petri dishes each containing 20 mL of spring water and the following larval densities: 1, 2, 4, 8, 16, 24, and 32. All larvae were counted into dishes at room temperature and then divided into the three different controlled environments, allowing a 12 h temperature acclimation period before predator introduction (Fig 1). For each of six combinations of copepod species and temperature, there were 35 Petri dishes containing a total of 435 newly-hatched *Ae*. *albopictus* larvae, with a total of 2,610 larvae used across all predator treatments and controls (S3 Fig). This sample size allowed for four replicates of each larval density and one predator-absent control at each density, for each combination of copepod species and temperature (S3 Fig).

The next day, the copepod predators held at three different temperatures were introduced to larval Petri dishes of matching controlled temperatures (Fig 1). The copepods were removed after a 6 h period of predation (Fig 1), which follows the schedule of similar experiments [41, 42]. Each copepod was preserved in 80% ethanol so that its body size could later be measured and matched to the larval count data. Immediately following the removal of the copepods, the number of surviving larvae in each Petri dish was counted and recorded. In addition, the body lengths of the 10 gravid females selected from each species of copepod, as well as the body lengths of all copepods included as predators in the functional response experiments, were measured from the front of the cephalosome to the end of the last urosomite [43].

### Design of predation efficiency experiment

**Fig 2:**
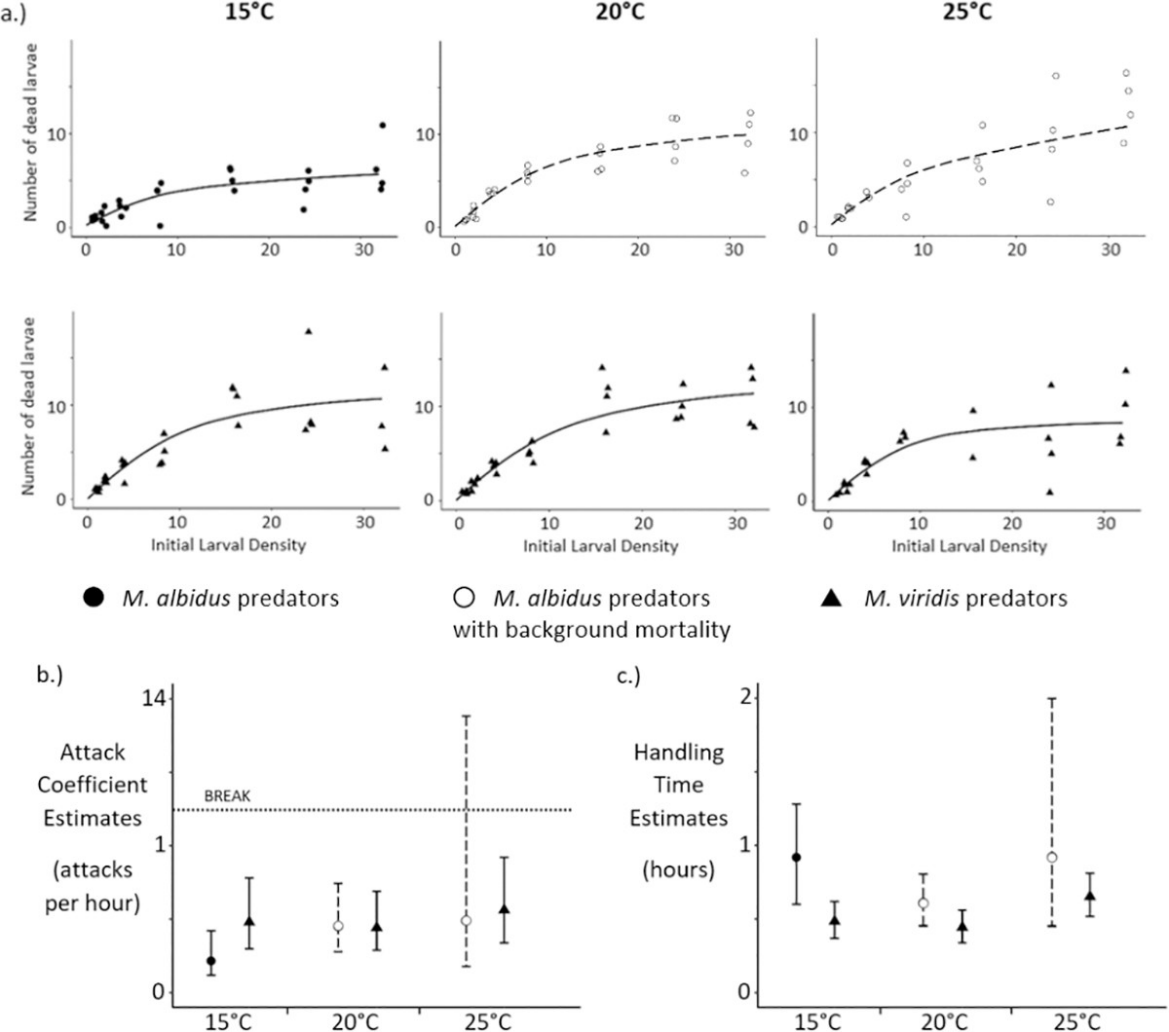
a.) Type II functional response curves for each combination of copepod species and temperature (Points are shown in the “jitter” position.) b.) Attack coefficient estimates and 95% confidence intervals by copepod species and temperature c.) Handling time estimates and 95% confidence intervals by copepod species and temperature.

In addition to functional response, predation efficiency has previously been measured to assess copepod predators of mosquito larvae [44]. We measured predation efficiency using a constant density of 24 larvae in 20 mL of spring water. This density was chosen from the higher end of the range of densities used in the functional response experiments, where the curves generally start to plateau (Fig 2A). The density where the functional response curves start to plateau represents the density at which the predators are satiated; increases in prey density after this point would not result in a higher number of prey killed by the predator [45].

*Ae*. *albopictus* larvae were hatched (S2 File) and any residual food was diluted in spring water following the same procedure used for the functional response experiments. Adult non-gravid female copepods were each placed in a Petri dish for a 24 h period of starvation and acclimation to three different temperature settings: 15 ± 1, 20 ± 1, and 25 ± 1°C, all at a 12:12 light/dark cycle (Fig 1). The largest non-gravid copepods of each species were selected to minimize the risk of selecting males or immature stages. The Petri dishes containing larvae were split into the three different temperature settings 12 h prior to the introduction of copepod predators, and there was a 6 h period of predation (Fig 1). At the end of the 6 h period, the copepods were removed, and each was stored individually in 80% ethanol so that predator body lengths could later be measured. The number of surviving larvae in each Petri dish was recorded immediately after removing the copepods.

At each of the three temperature settings, there was a total of 24 Petri dishes, each containing 24 larvae (1,728 larvae across all temperatures); the 24 dishes were divided into three groups (n = 8): one with *M*. *albidus* predation, one with *M*. *viridis* predation, and one as a control (S3 Fig). Every predator treatment was matched to a control that had been held at the same temperature (S3 Fig). Each temperature setting had a total of 192 control larvae. Larval background mortality was 5% at 15°C, 11% at 20°C, and 3% at 25°C. A total of 24 *M*. *albidus* and 24 *M*. *viridis* copepods were used in this experiment.

### Functional response curve analysis

The general shapes of the functional response curves were determined for each combination of copepod species and temperature using predator-present data. When polynomial logistic functions are fitted to describe the relationship between the proportion of larvae killed and the initial larval density, a negative first-order term indicates a type II response, and a positive first-order term indicates a type III response [45, 46]. The “frair_test” function (“frair” package, R version 3.4.2) was used to determine the shapes of the functional response curves according to the sign and significance of first-order and second-order terms in logistic regressions [46, 47]. Three observations were excluded from the *M*. *albidus* at 25°C group—two due to copepod deaths during the starvation period, and one because two copepod predators were introduced into the same Petri dish. Three observations were excluded from the *M*. *viridis* at 25°C group, and one was excluded from the *M*. *viridis* at 15°C group; all four *M*. *viridis* exclusions were due to insufficient body length of the copepod indicating that these predators could have been either males or immatures, rather than adult non-gravid females.

Mortality of *Ae*. *albopictus* larvae was observed in controls for five out of the six combinations of copepod species and temperature. *M*. *viridis* predators at 25°C was the only group without larval mortality in any of the controls. For *M*. *albidus*, across all control larvae (n = 87) at 15°C, three deaths were observed (3% mortality); four deaths were observed at 20°C (5% mortality); and eleven deaths were observed at 25°C (13% mortality). For *M*. *viridis*, two deaths were observed among control larvae at 15°C (2% mortality), and three deaths were observed at 20°C (3% mortality). Mortality rates of aedine larvae in predator-absent controls have previously been observed to be as high as 20% [44]. To account for both background mortality and prey depletion caused by predation, the functional response parameters (attack coefficient and handling time) were estimated by fitting the following differential equation:

$$\frac{dN}{dt}=-\frac{bN^{1+q}}{1+bhN^{1+q}}\;P-mN$$ Eq 1

where *dN/dt* is the change in prey density over time, *b* is the attack coefficient, *q* is an exponent that can fall between a type II response (q = 0) and a type III response (q = 1), *h* is the handling time, *P* is the predator density, and *m* is the mortality rate [48].

Since background prey mortality should not alter the general shape of the functional response curve [48], the *q* parameter was fixed to either 0 or 1 based on the results of the “frair_test” function, which only considers data from predator-present treatments [46, 47]. The attack coefficient (*b*) was given a starting value of one, the handling time (*h*) had a starting value equivalent to the inverse of the maximum feeding rate, and the mortality rate (*m*), if applicable, was given a starting value of 0.01 [48]. The attack coefficient and handling time parameters were estimated based on our empirical data using an iterative maximum likelihood method: “mle2” function, “bbmle” package, R version 3.4.2 [48–50].

The “nll.ode.general.mort” function [48] was used to fit data that included some background prey mortality to the statistical model (Eq 1). In cases where the mortality rate was shown to be insignificant, or where no larval mortality was originally observed, the “nll.bolker” function [48], which assumes a mortality rate of zero, was used. Both functions are negative log-likelihood functions that are minimized by applying maximum likelihood estimation methods [48]. Confidence intervals for the model parameters of interest, attack coefficient and handling time, were computed by the “confint” function, base package, R version 3.4.2. To detect differences in functional response parameter estimates due to either temperature category or copepod species, 95% confidence intervals were compared; this method for comparing parameter estimates across temperatures has previously been used in a similar analysis of copepod predation [51].

### Predation efficiency analysis

For each pair of predator treatment and control (S3 Fig), the copepod’s predation efficiency was calculated according to Abbott’s formula [44, 52]:

$$Predation\;efficiency=\frac{Number\;alive\;in\;control-Number\;alive\;in\;treatment}{Number\;alive\;in\;control}\;(100)$$ Eq 2

Copepod body lengths, measured in mm, were converted to estimates of body mass (mg) using an equation from previous studies [51, 53, 54]:

$$Mass=0.055\;x\;length^{2.73}$$ Eq 3

Two linear regression models were fitted to explain predation efficiency. The first tested temperature and copepod species as predictors, and the second tested copepod body mass in place of species (S3 File). Both models were also fitted without temperature predictors (S3 File).

### Body mass differences by copepod species and experimental design

Within each experimental design (functional response and predation efficiency), the distribution of body masses was assessed for each copepod species using the Shapiro-Wilk test for normality. Based on these results, either a Wilcoxon rank sum test or a Welch two sample t-test was used to determine if the body masses differed due to copepod species.

Among the predators used in the functional response experiments, each *M*. *albidus* copepod was randomly paired with an *M*. *viridis* copepod (80 pairs). Predation efficiency predators were also randomly paired, yielding 23 pairs of the two species. For each pair, the *M*. *albidus* body mass was subtracted from that of *M*. *viridis*, generating 103 differences in body mass due to species. A Wilcoxon rank sum test was used to determine if the body mass differences between copepod species varied by experimental design.

Analyses were completed in R version 3.4.2, and all data will be made accessible from the Dryad Digital Repository.

## Results

### Functional response curves

A type II functional response, demonstrated through logistic regression results (S1 Table), was observed in every combination of copepod species and temperature (Fig 2A). There was a significant effect of background mortality on the change in prey density over time for *M*. *albidus* at 20 and 25°C (S2 Table). Within each copepod species, there was no significant difference in either attack coefficient or handling time due to temperature (Fig 2, S4 Table). Within each temperature setting, there was no significant difference in either type of parameter estimate due to copepod species (Fig 2, S4 Table). The 95% confidence interval around the attack coefficient estimate for *M*. *albidus* at 25°C was extremely wide, representing high uncertainty (Fig 2B). This imprecise estimate was likely due to the highly significant effect of mortality rate (p-value = 0.0040) on the change in prey density over time (S2 Table). In addition, the mortality rate’s standard error was higher for *M*. *albidus* at 25°C than it was for any other data subset that included background larval mortality (S2 Table), and the model deviance (-2 log L = 111.25) was higher than that of any other model used to generate parameter estimates (S2 and S3 Tables).

### Predation efficiency

Predation efficiency values ranged from 5.0% to 58.3%, with a median of 21.7%, and a slightly right-skewed distribution (skewness = 0.61, kurtosis = 2.9).

**Fig 3:**
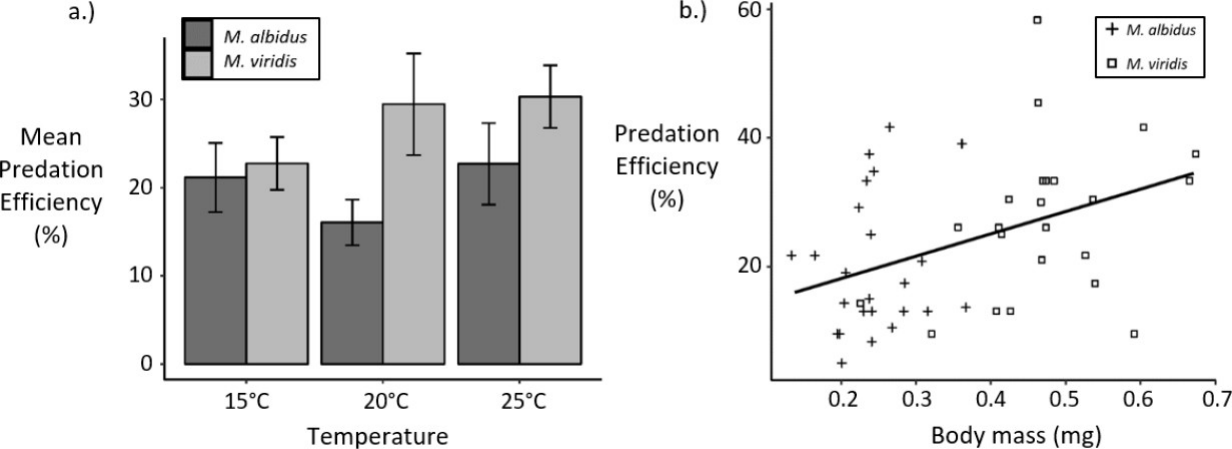
a.) Bar chart of predation efficiency by copepod species and temperature (Error bars represent ± the standard error.) b.) Predation efficiency by copepod body mass (Points are shown in the “jitter” position).

A linear model testing copepod species and temperature category as predictors of predation efficiency explained 6.8% of the variance in predation efficiency (Table 1). The predation efficiency of *M*. *viridis* copepods was 25.7%, significantly higher (p-value = 0.0288) than that of *M*. *albidus* copepods, which had a predation efficiency of 18.2%, when controlling for differences in temperature (Table 1, Fig 3A). Interactions between species and temperature were not significant (Species x Temperature_20_, p-value = 0.150; Species x Temperature_25_, p-value = 0.466).

**Table 1:** Linear regression of predation efficiency by copepod species and temperature (n = 47).

Parameter	Estimate	Standard Error	p-value	Adjusted R^2^	AIC
Intercept	18.19	3.30	1.92 x 10^−6^	0.068	373.1
Species (*M*. *viridis*, ref = *M*. *albidus*)	7.54	3.33	0.0288		
Temperature (20°C, ref = 15°C)	0.80	4.04	0.8432		
Temperature (25°C, ref = 15°C)	4.56	4.10	0.2731		

A linear model testing copepod body mass and temperature category as predictors of predation efficiency explained 14.0% of the variance in predation efficiency (Table 2). For each 0.1 mg increase in copepod body mass, the predation efficiency increased by approximately 3.4 percentage points (p-value = 0.0042), when controlling for differences in temperature (Table 2, Fig 3B). Interactions between body mass and temperature were not significant (Mass x Temperature_20_, p-value = 0.611; Mass x Temperature_25_, p-value = 0.565).

**Table 2:** Linear regression of predation efficiency by copepod body mass and temperature (n = 47).

Parameter	Estimate	Standard Error	p-value	Adjusted R^2^	AIC
Intercept	9.72	4.88	0.0528	0.140	369.3
Body Mass (mg)	34.44	11.37	0.0042		
Temperature (20°C, ref = 15°C)	0.83	3.88	0.8305		
Temperature (25°C, ref = 15°C)	4.04	3.94	0.3106		

For both the species model (Table 1) and the body mass model (Table 2), removing the temperature predictors increased the adjusted R^2^ value and decreased the AIC (Table 3, S5 and S6 Tables).

**Table 3:** Model selection.

Parameters	Degrees of Freedom	Adjusted R^2^	AIC^a^
Species, Temperature_20_, Temperature_25_	43	0.068	373.1
Species, Temperature_20_, Temperature_25_, Species x Temperature_20_, Species x Temperature_25_	41	0.071	390.7
Species	45	0.081	362.6
Mass, Temperature_20_, Temperature_25_	43	0.140	369.3
Mass, Temperature_20_, Temperature_25_, Mass x Temperature_20_, Mass x Temperature_25_	41	0.107	388.8
Mass	45	0.156	358.5

a.) A difference in AIC value greater than or equal to 2 is the suggested minimum difference for model selection purposes [55].

### Body mass differences by copepod species and experimental design

*M*. *viridis* copepods were larger than *M*. *albidus* copepods in both the functional response (p-value < 0.0001) and the predation efficiency (p-value < 0.0001) experiments, and both copepod species were larger in the predation efficiency experiments than in the functional response experiments (S4 Fig, S4 File).

The body mass difference between copepod species was greater in the predation efficiency experiments than in the functional response experiments (p-value = 0.0136). In the predation efficiency experiments, the median increase from *M*. *albidus* to *M*. *viridis* was 0.24 mg, while in the functional response experiments, the median increase was 0.12 mg.

## Discussion

Both *M*. *albidus* and *M*. *viridis* copepods collected from Surrey, UK would be appropriate for use in an inundative release biocontrol program because both predator species display type II functional response curves when presented with invasive *Ae*. *albopictus* larvae (S1 Table). Type II functional response curves are preferred because they demonstrate the predator’s ability to eliminate invasive populations at low prey densities [33]. Other studies using *M*. *albidus* and *M*. *viridis* from Northern Ireland against mosquito larvae prey have also found evidence of type II functional response curves [18, 19].

Our functional response analyses did not detect any differences in response type, attack coefficient, or handling time due to copepod species or temperature, tested at 15, 20, and 25°C (Fig 2, S1 Table). In addition, we found that temperature categorical variables were not significant predictors of copepod predation efficiency against *Ae*. *albopictus* larvae (Tables 1–3). A meta-analysis of 648 functional responses from 86 different studies found that although attack rate increased with temperature, the increase was less steep than that which had been expected based on metabolic theory [56]. In addition, a previous study of predator-prey interactions involving sit-and-wait copepod predators found no significant difference in the attack parameter of the functional response across three different temperatures: 18, 22, and 26°C [51]. Since the effect of temperature on sit-and-wait predation is driven mainly by the velocity of the prey [57], our results suggest that the velocity of newly-hatched *Ae*. *albopictus* larvae does not change dramatically over a temperature range of 15–25°C. From a public health perspective, the absence of significantly lower predation efficiency at lower temperatures is beneficial because *Ae*. *albopictus* larvae that develop at lower temperatures have been shown to be more susceptible to chikungunya infection as adults [58].

The term “handling time” has been defined differently by different studies. For example, one study defined it as “the time lapsed between an attack of the prey by the copepod and its resumption of movement after ingestion,” and found that *M*. *thermocyclopoides* copepods displayed an average handling time of 83 s for first instar *Anopheles stephensi* larvae and 185 s for first instar *Cx*. *quinquefasciatus* [14]. These times are consistent with directly-observed ingestion times reported by another study, which states that a mosquito larva is usually consumed by a cyclopoid copepod within a few minutes [28]. However, the original methods designed for fitting functional response curves assume that the predator only has “two time-consuming behaviours–searching and handling of prey” [31]. In this context, the “handling time” has been defined as “the time lost from searching per resource consumed,” not as the ingestion time per resource consumed [37]. Our handling time estimates (Fig 2C, S4 Table) are based on the original framework of functional response analysis, and therefore represent average predator search times per larva consumed. The prey-searching techniques of *M*. *albidus* and *M*. *viridis* have previously been described as “[relying] on bumping into … prey during the course of … meanderings” [59]. This description of rather inefficient prey searching is consistent with our handling time estimates of approximately 30 min to 1 h (Fig 2C, S4 Table).

We found that *M*. *viridis* was a significantly more efficient predator of *Ae*. *albopictus* than *M*. *albidus* (Table 1, Fig 3, and S5 Table). An analysis of the gut contents of English Lake District copepods previously found that 15.7% of *M*. *viridis* had consumed dipterous larvae, compared to only 7.4% of *M*. *albidus* [60]. Thus, it is possible that English *M*. *viridis* may be better adapted to feeding on mosquito larvae than English *M*. *albidus*. A study using *M*. *albidus* and *M*. *viridis* from Northern Ireland against *Culex* mosquitoes from Surrey, UK suggested that *M*. *albidus* is more effective than *M*. *viridis* as a biocontrol [19]. It appears this is not the case for invasive *Ae*. *albopictus* prey. A previous study showed that an aedine species (*Ae*. *aegypti*) was more vulnerable to predation by *M*. *viridis* than *Cx*. *pipiens*, despite the larger head capsule width of *Ae*. *aegypti* [61]. In addition, *M*. *viridis* was among three cyclopoid copepod species from Northern Ireland that displayed a preference for *Ae*. *albopictus* larvae over *Cx*. *pipiens* prey [18]. Another study of *M*. *albidus* and *M*. *viridis* from Northern Ireland as predators of both *Paramecium caudatum* and *Cx*. *pipiens* reported that *M*. *viridis* consumed more prey organisms overall than *M*. *albidus* [42].

The larger size of *M*. *viridis* contributes to the higher predation efficiency observed among that species against *Ae*. *albopictus* (Fig 3B). Our analysis shows that copepod body mass was a better predictor of predation efficiency than species identity (Tables 1–3). The linear regression model with the highest adjusted R-squared value, 16%, was the model with copepod body mass as the sole predictor of predation efficiency (Table 3, S6 Table). The positive relationship we observed between copepod body mass and predation efficiency (Table 2, Fig 3B, and S6 Table) is consistent with the findings of a previous meta-analysis on crustacean predation of immature fish, which showed that the predation rate was negatively related to the prey/predator size ratio [62]. In addition, a study specific to mosquito prey found that consumption rate increased with predator body mass when comparing three different predators of mosquitoes: copepods, dragonfly naiads, and mosquitofish [63]. Our data are also in agreement with the positive linear relationship between body mass and ingestion rate that has been demonstrated in other taxa, including herbivorous and carnivorous endotherms, as well as carnivorous poikilothermic tetrapods [64]. Although a recent study using *Ae*. *japonicus* larvae as prey found that *M*. *viridis* predation rates began to decrease when predators exceeded 1.8 mm in length [65], our data represent copepods of up to 2.5 mm in length, and there is no evidence of lower predation efficiency among the largest predators (Fig 3B). According to the theory that, at equilibrium, “the rate of food intake is equal to the rate at which food is leaving the gut” [66], our results suggest that *M*. *viridis* may have a higher gut clearance rate than *M*. *albidus*, possibly due to its larger size.

Despite the higher predation efficiency observed among *M*. *viridis* (Table 1, Fig 3, and S5 Table), we did not detect any differences between the two copepod species in their functional response parameter estimates, when controlling for temperature (Fig 2, S4 Table); this was perhaps because the predation efficiency experiment had a larger sample size of observations for each combination of predictors, and therefore more power to detect differences. In addition, while the size difference between *M*. *albidus* and *M*. *viridis* was significant in both experimental designs, there was a greater difference in size due to species among the copepods used in the predation efficiency experiment than among the copepods used in the functional response experiment (S4 Fig, S4 File).

Although we did not detect any difference in functional response parameter estimates or predation efficiency due to temperature, the temperature at which copepods are cultured impacts their size [21], and our results show that copepod size is a determinant of their predation efficiency. A previous study, in which UK *M*. *albidus* and *M*. *viridis* were cultured at six different temperatures between 5 and 20°C, showed a consistent negative relationship between adult body mass of both species and culturing temperature [21]. Evidence of this relationship between size and rearing temperature had already been recorded for freshwater copepods collected in Paris [67]. Although cultures tend to have higher rates of reproduction at higher temperatures [21], we recommend that the importance of individual body mass be taken into consideration when determining the optimal temperature for mass copepod culturing.

Fecundity data from older studies [21, 68] have recently been incorporated into metrics used to compare the efficacy of different copepod species as biocontrol agents [18, 19]. Caution is recommended when interpreting these metrics if the fecundity variable is either based on an inadequate number of observations or derived from multiple populations of copepods. For example, a previous calculation used to determine the “reproductive effort” of *M*. *viridis* incorporated a clutch weight value based on the dimensions of a single egg sac from a female collected at an unrecorded temperature in South Germany, female body weight data from a population in South Germany, and embryonic development time data from English Lake District copepods held at 20°C [68, 69]. This reproductive effort value was then applied to calculate a metric for *M*. *viridis* from Northern Ireland that were cultured at 25°C [18]. While it is time-consuming to collect robust and relevant fecundity data experimentally, biocontrol metrics can only be meaningful if they are based on appropriate original data.

Life history characteristics other than fecundity, particularly lifespan and resistance to starvation, also impact the efficiency of biocontrol agents, and these characteristics have been shown to positively correlate with cyclopoid copepod body size [68, 70]. The relationship between lifespan and body size is recognized as a general rule in ecology and has been exemplified by comparing the lifespan of an elephant to that of a mouse [71]. *M*. *viridis* females from English Lake District cultures have previously been observed to spend approximately 120 days (at 15°C) and 80 days (at 20°C) in the adult life stage [21]. The fecundity and long-term stability of field copepod populations can be difficult to predict. A year-round field study conducted in Florida, USA of cyclopoid copepod populations in tire piles found that the number of copepods observed was influenced by the age and amount of leaf litter in each tire [72]. In addition, cyclopoid copepod populations survive longer in tires near shade or vegetation because the water in the tire is less likely to evaporate [73]. In the UK, where *Ae*. *albopictus* adults are only expected to be active from May to September [5], the stability of year-round copepod populations in tires may not be a priority. The use of copepods as biocontrol agents in UK field sites might benefit from bi-monthly re-applications of well-maintained laboratory cultures, and perhaps more frequent reinforcements in case of long droughts followed by substantial precipitation. A control strategy that is based on precipitation patterns and the estimated survival time of adult copepods in the field would be less risky than a strategy that relies on the viability of subsequent copepod generations.

As the invasion of *Ae*. *albopictus* into the UK progresses, it may become more relevant to conduct predation experiments on mosquito larvae that are hatched from colonies acclimated to lower temperatures. In our experiments, the *Ae*. *albopictus* larvae were from a colony that was reared under conditions experienced on warm summer days in Montpellier, France. This approach is currently valid because *Ae*. *albopictus* has not yet established in the UK and eggs that could be introduced via used tire shipments would be naïve to UK weather conditions. However, in future decades, it may be more appropriate to use *Ae*. *albopictus* larvae from a colony reared under conditions reflective of the diurnal temperature range experienced in the southern UK.

## Supporting information

S1 Fig Tire locations. (PDF)

S2 Fig May through September of 2018 weekly tire water temperatures. (PDF)

S3 Fig Experimental design: (a) functional response repeated six times, once for each combination of predator species and temperature; (b) predation efficiency repeated three times, once for each temperature.
(PDF)

S4 Fig Boxplot of copepod body mass by experimental design. (PDF)

S1 Table Results of logistic regression of proportional consumption data as a function of initial prey density using the “frair_test” function. (PDF)

S2 Table Results of fitting the “nll.ode.general.mort” function for functional response curves in which background mortality was observed. (PDF)

S3 Table Results of fitting the “nll.bolker” function for functional response curves in which background mortality was either not observed or insignificant. (PDF)

S4 Table Functional response parameter estimates and 95% confidence intervals. (PDF)

S5 Table Linear regression of predation efficiency by copepod species (n = 47). (PDF)

S6 Table Linear regression of predation efficiency by copepod body mass (n = 47). (PDF)

S1 File Collection of field temperature data. (PDF)

S2 File *Ae*. *albopictus* hatching procedure for functional response and predation efficiency experiments. (PDF)

S3 File Predation efficiency linear regression models. (PDF)

S4 File Copepod body sizes by species and experimental design. (PDF)
