# Supplementary material for: Size, not temperature, drives cyclopoid copepod predation of invasive mosquito larvae
Source: PLoS One. 2021 Feb 2;16(2):e0246178. doi: 10.1371/journal.pone.0246178 (PMC7853444; doi:10.1371/journal.pone.0246178)
Supplement: S1 Fig — (PDF) [file pone.0246178.s001.pdf]

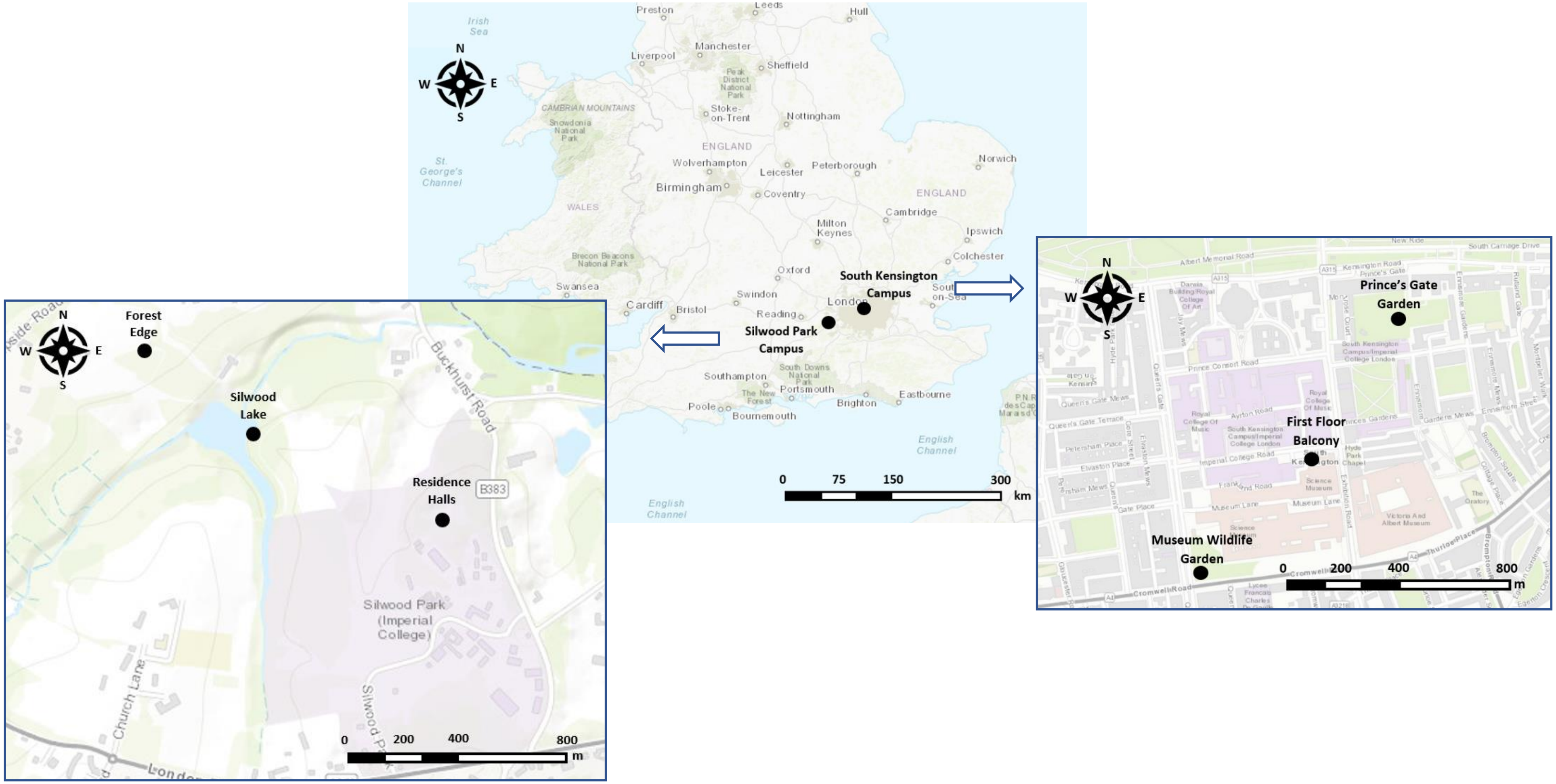

S1 Fig. Tire Locations (Topographic base map was accessed from USGS National Map Viewer: https://viewer.nationalmap.gov/advanced-viewer/)
