# Supplementary material for: Size, not temperature, drives cyclopoid copepod predation of invasive mosquito larvae
Source: PLoS One. 2021 Feb 2;16(2):e0246178. doi: 10.1371/journal.pone.0246178 (PMC7853444; doi:10.1371/journal.pone.0246178)
Supplement: S2 Fig — (PDF) [file pone.0246178.s002.pdf]

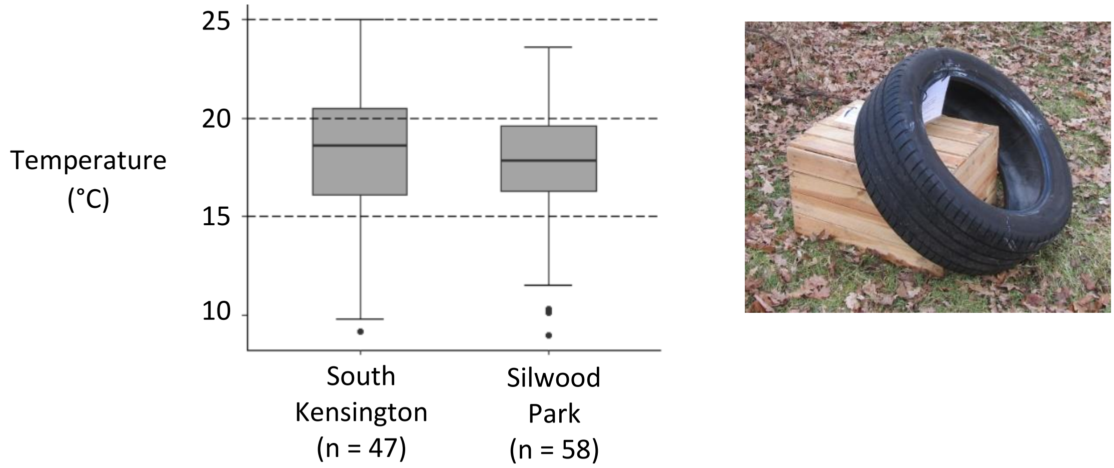

S2 Fig. May through September of 2018 weekly tire water temperatures (Dotted lines represent the three temperatures tested in the functional response and predation efficiency experiments.)
