# Supplementary material for: Size, not temperature, drives cyclopoid copepod predation of invasive mosquito larvae
Source: PLoS One. 2021 Feb 2;16(2):e0246178. doi: 10.1371/journal.pone.0246178 (PMC7853444; doi:10.1371/journal.pone.0246178)
Supplement: S3 Fig — Experimental design: (a) functional response repeated six times, once for each combination of predator species and temperature; (b) predation efficiency repeated three times, once for each temperature. (PDF) [file pone.0246178.s003.pdf]

a.)

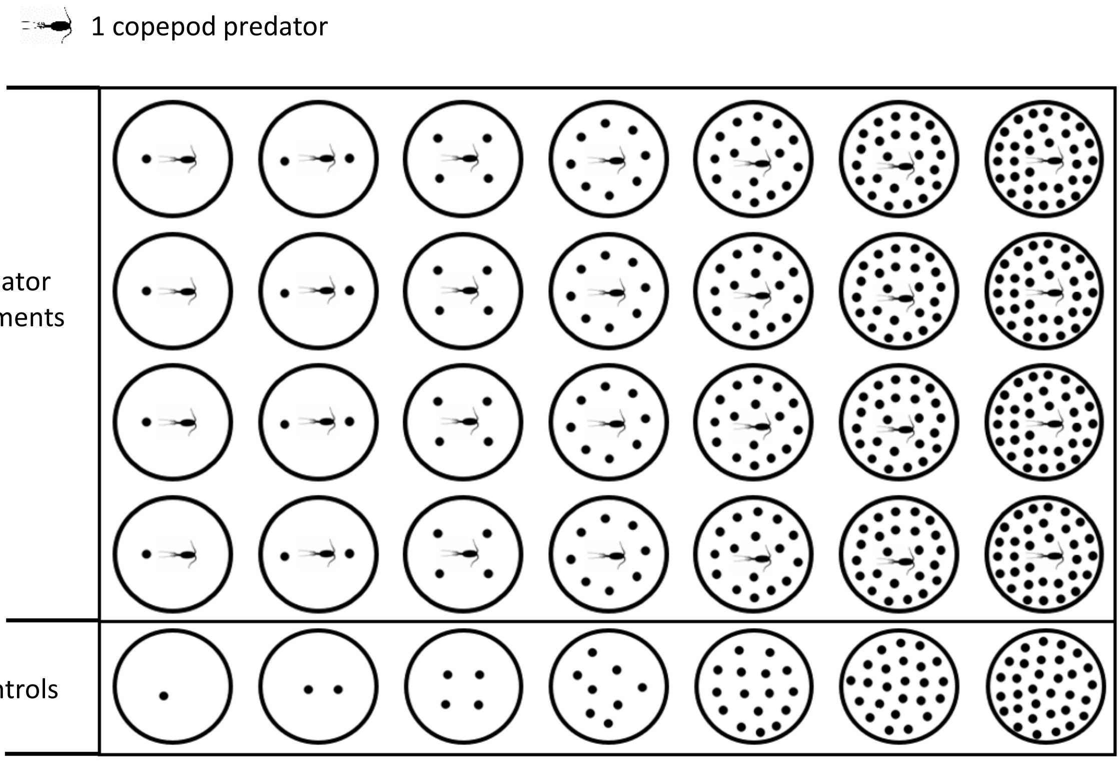

b.)

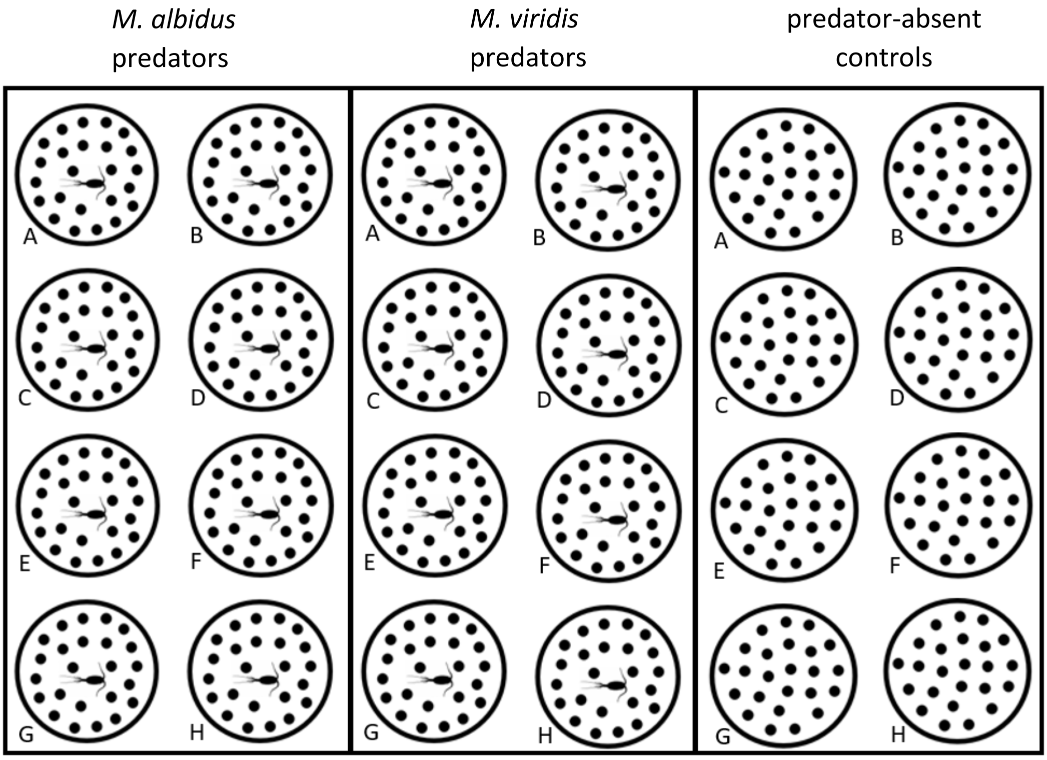

S3 Fig. Experimental design: (a) functional response repeated six times, once for each combination of predator species and temperature; (b) predation efficiency repeated three

times, once for each temperature (Source of copepod silhouette accessed August 2019: http://phylopic.org/name/f79c5b86-7b73-468a-9ad4-995646398f99)
