# Supplementary material for: Size, not temperature, drives cyclopoid copepod predation of invasive mosquito larvae
Source: PLoS One. 2021 Feb 2;16(2):e0246178. doi: 10.1371/journal.pone.0246178 (PMC7853444; doi:10.1371/journal.pone.0246178)
Supplement: S4 Fig — (PDF) [file pone.0246178.s004.pdf]

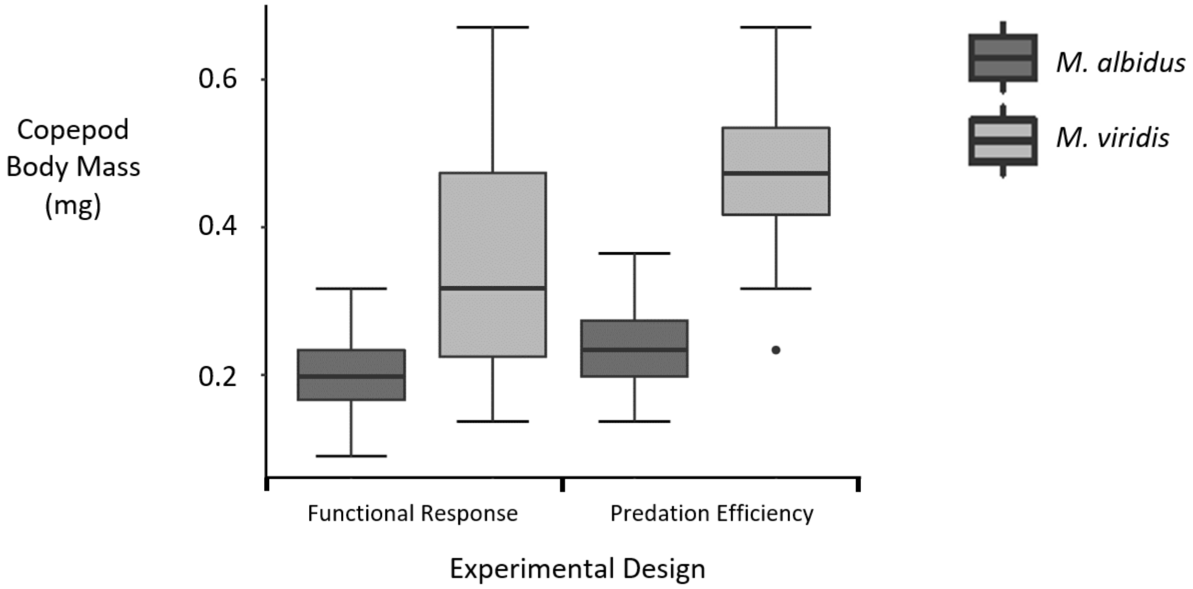

S4 Fig. Boxplot of copepod body mass by experimental design (Sample sizes: Functional response, *M. albidus* = 81; Functional response, *M. viridis* = 80; Predation efficiency, *M. albidus* = 24; Predation efficiency, *M. viridis* = 23.)
