# Supplementary material for: Size, not temperature, drives cyclopoid copepod predation of invasive mosquito larvae
Source: PLoS One. 2021 Feb 2;16(2):e0246178. doi: 10.1371/journal.pone.0246178 (PMC7853444; doi:10.1371/journal.pone.0246178)
Supplement: S1 Table — (PDF) [file pone.0246178.s005.pdf]

S1 Table. Results of logistic regression of proportional consumption data as a function of initial prey density using the “frair_test” function

**Species**	**Temperature (°C)**	**n**[a]	**First Order Term**[b]	**p - value**
M. albidus	15	28	-0.06	<0.0001
M. albidus	20	28	-0.08	<0.0001
M. albidus	25	25	-0.04	0.0007
M. viridis	15	27	-0.09	<0.0001
M. viridis	20	28	-0.08	<0.0001
M. viridis	25	25	-0.09	<0.0001

a.) Controls are not included for determining the type of functional response.

b.) The response is classified as “type II” when there is a significant negative first-order term.
