# Supplementary material for: Size, not temperature, drives cyclopoid copepod predation of invasive mosquito larvae
Source: PLoS One. 2021 Feb 2;16(2):e0246178. doi: 10.1371/journal.pone.0246178 (PMC7853444; doi:10.1371/journal.pone.0246178)
Supplement: S2 Table — (PDF) [file pone.0246178.s006.pdf]

S2 Table. Results of fitting the "nll.ode.general.mort" function for functional response curves in which background mortality was observed

**Species**	**Temp. (°C)**	**n[a]**	**Parameter**	**Estimate**	**Standard Error**	**p-value**	**-2 log L**
M. albidus	15	35	attack coefficient	0.2379	0.0921	0.0098	102.73
			handling time	1.1583	0.2782	<0.0001	
			mortality rate	0.0063	0.0036	0.0816	
M. albidus	20	35	attack coefficient	0.4561	0.1156	<0.0001	90.98
			handling time	0.6036	0.0838	<0.0001	
			mortality rate[2]	0.0076	0.0038	0.0426	
M. albidus	25	32	attack coefficient	0.4909	0.3523	0.1634	111.25
			handling time	0.9163	0.3601	0.0109	
			mortality rate[b]	0.0289	0.0100	0.0040	
M. viridis	15	34	attack coefficient	0.4911	0.1214	<0.0001	100.14
			handling time	0.5163	0.0705	<0.0001	
			mortality rate	0.0038	0.0027	0.1569	
M. viridis	20	35	attack coefficient	0.4511	0.1063	<0.0001	93.30
			handling time	0.4848	0.0667	<0.0001	
			mortality rate	0.0057	0.0033	0.0823	

a.) Number of empirical observations includes both predator and control treatments.

b.) Background prey mortality had a significant impact on fitting the model for *M. albidus* at 20 and 25°C.
