# Supplementary material for: Size, not temperature, drives cyclopoid copepod predation of invasive mosquito larvae
Source: PLoS One. 2021 Feb 2;16(2):e0246178. doi: 10.1371/journal.pone.0246178 (PMC7853444; doi:10.1371/journal.pone.0246178)
Supplement: S3 Table — (PDF) [file pone.0246178.s007.pdf]

S3 Table. Results of fitting the "nll.bolker" function for functional response curves in which background mortality was either not observed or insignificant

Species	Temp. (°C)	n	Parameter	Estimate	Standard Error	p-value	-2 log L
M. albidus	15	28	attack coefficient	0.2166	0.0716	0.0025	89.71
			handling time	0.9189	0.1696	<0.0001	
M. viridis	15	27	attack coefficient	0.4803	0.1137	<0.0001	92.14
			handling time	0.4853	0.0619	<0.0001	
M. viridis	20	28	attack coefficient	0.4430	0.0989	<0.0001	83.57
			handling time	0.4447	0.0554	<0.0001	
M. viridis	25	25	attack coefficient	0.5629	0.1438	<0.0001	85.53
			handling time	0.6527	0.0725	<0.0001	
