# Supplementary material for: Size, not temperature, drives cyclopoid copepod predation of invasive mosquito larvae
Source: PLoS One. 2021 Feb 2;16(2):e0246178. doi: 10.1371/journal.pone.0246178 (PMC7853444; doi:10.1371/journal.pone.0246178)
Supplement: S4 Table — (PDF) [file pone.0246178.s008.pdf]

S4 Table. Functional response parameter estimates and 95% confidence intervals

**Species**	**Temp. (°C)**	**Attack Coefficient (attacks per hour)**	**Handling Time (hours)**
M. albidus	15	0.217, (0.116 – 0.425)	0.919, (0.596 – 1.28)
M. albidus	20	0.456, (0.277 – 0.747)	0.604, (0.452 – 0.798)
M. albidus	25	0.491, (0.178 – 13.9)	0.916, (0.451 – 1.99)
M. viridis	15	0.480, (0.305 – 0.777)	0.485, (0.368 – 0.615)
M. viridis	20	0.443, (0.287 – 0.691)	0.445, (0.337 – 0.559)
M. viridis	25	0.563, (0.338 – 0.921)	0.653, (0.519 – 0.808)
