# Supplementary material for: Size, not temperature, drives cyclopoid copepod predation of invasive mosquito larvae
Source: PLoS One. 2021 Feb 2;16(2):e0246178. doi: 10.1371/journal.pone.0246178 (PMC7853444; doi:10.1371/journal.pone.0246178)
Supplement: S5 Table — (PDF) [file pone.0246178.s009.pdf]

S5 Table. Linear regression of predation efficiency by copepod species (n = 47)

**Parameter**	**Estimate**	**Standard Error**	**p-value**	**Adjusted R^2**	**AIC**
Intercept	19.97	2.314	4.25×10^{-11}	0.081	362.6
Species (*M. viridis*, ref = *M. albidus*)	7.42	3.308	0.0299		
