# Supplementary material for: Size, not temperature, drives cyclopoid copepod predation of invasive mosquito larvae
Source: PLoS One. 2021 Feb 2;16(2):e0246178. doi: 10.1371/journal.pone.0246178 (PMC7853444; doi:10.1371/journal.pone.0246178)
Supplement: S6 Table — (PDF) [file pone.0246178.s010.pdf]

S6 Table. Linear regression of predation efficiency by copepod body mass (n = 47)

**Parameter**	**Estimate**	**Standard Error**	**p-value**	**Adjusted R^2**	**AIC**
Intercept	11.19	4.33	0.0130	0.156	358.5
Body Mass (mg)	34.75	11.26	0.0035		
