# Supplementary material for: Size, not temperature, drives cyclopoid copepod predation of invasive mosquito larvae
Source: PLoS One. 2021 Feb 2;16(2):e0246178. doi: 10.1371/journal.pone.0246178 (PMC7853444; doi:10.1371/journal.pone.0246178)
Supplement: S1 File — (PDF) [file pone.0246178.s011.pdf]

S1 File: Collection of field temperature data

In late February and early March of 2018, six empty car tires, each leaning on a wooden crate, were placed in six different locations (S1 Fig), spread between two main sites: South Kensington (London, UK) and Silwood Park (Berkshire, UK). Although most tires were at ground-level near vegetation or shade, the "First Floor Balcony" tire (S1 Fig) had more direct sun exposure, and thus, may be more representative of the conditions present in large tire piles. Each tire was surveyed once a week from the 16th of April until the 30th of October between 11am and 5pm, which matches the time period of predation for both our functional response and predation efficiency experiments. If water was present in a tire, the water temperature was taken using a hand-held Hanna Instruments thermometer (model name: "Checktemp 1"). Temperature data from Silwood Park and South Kensington were recorded from May through September of 2018 (S2 Fig), the months of predicted *Ae. albopictus* adult activity [1]. There were 105 observations in total; 16 temperature recordings were missing because the tire was dry, and two were excluded because they were recorded outside the time period of 11am to 5pm.

Reference

1. Medlock JM, Avenell D, Barrass I, Leach S. Analysis of the potential for survival and seasonal activity of Aedes albopictus (Diptera : Culicidae) in the United Kingdom. Journal of Vector Ecology. 2006;31(2):292-304.
