# Supplementary material for: Size, not temperature, drives cyclopoid copepod predation of invasive mosquito larvae
Source: PLoS One. 2021 Feb 2;16(2):e0246178. doi: 10.1371/journal.pone.0246178 (PMC7853444; doi:10.1371/journal.pone.0246178)
Supplement: S2 File — (PDF) [file pone.0246178.s012.pdf]

S2 File: *Ae. albopictus* hatching procedure for functional response and predation efficiency experiments

Ae. albopictus eggs were collected from the colony on filter papers and stored in plastic bags containing damp paper towels to maintain humidity. Stored egg papers were submerged in 3 mg/L nutrient broth solution (Sigma-Aldrich © 70122 Nutrient Broth No 1) and oxygen was displaced by vacuum suction for 30 min. Immediately following oxygen displacement, ground fish food (Cichlid Gold Hikari®, Japan) was added *ad libitum*. The eggs were left in this solution at 27 ± 1°C for 12 h [1] before the newly-hatched larvae were counted (Fig 1). The hatching temperature was kept high, relative to the experimental temperatures (15-25°C), to maximize the hatch rate over a short, semi-synchronous time period, especially since *Ae. albopictus* often hatch in multiple installments [1].

Reference

1. Hanson SM, Craig GB, Jr. Cold acclimation, diapause, and geographic origin affect cold hardiness in eggs of Aedes albopictus (Diptera: Culicidae). J Med Entomol. 1994;31(2):192-201.
