# Supplementary material for: Size, not temperature, drives cyclopoid copepod predation of invasive mosquito larvae
Source: PLoS One. 2021 Feb 2;16(2):e0246178. doi: 10.1371/journal.pone.0246178 (PMC7853444; doi:10.1371/journal.pone.0246178)
Supplement: S3 File — (PDF) [file pone.0246178.s013.pdf]

S3 File: Predation efficiency linear regression models

The following linear regression model was fitted to test temperature setting and copepod species as predictors of predation efficiency:

$$Y = \beta_0 + \beta_1(\text{Species}) + \beta_2(\text{Temperature}_{20}) + \beta_3(\text{Temperature}_{25}) + \varepsilon \qquad \text{eqn S1}$$

where Y represents the predation efficiency; β_0 is the Y-intercept; Species is a binary variable denoted as 0 for *M. albidus* and 1 for *M. viridis*; Temperature_{20} is a binary variable denoted as 0 for 15°C and 25°C, and 1 for 20°C; Temperature_{25} is a binary variable denoted as 0 for 15°C and 20°C, and 1 for 25°C; and ε is a random error term, assumed $\sim N(0, \sigma^2)$. Forty-seven observations were included in the model; one was excluded because the copepod predator (*M. viridis* at 25°C) was found to have died at some point during the 6 h predation period. The statistical significance of each independent parameter estimate was analyzed at $\alpha = 0.05$.

An additional linear regression model was fitted to test temperature setting and copepod body mass as predictors of predation efficiency:

$$Y = \beta_0 + \beta_1(\text{Mass}) + \beta_2(\text{Temperature}_{20}) + \beta_3(\text{Temperature}_{25}) + \varepsilon \qquad \text{eqn S2}$$

where Mass is a continuous variable referring to each copepod's body mass in mg; and all other notation is identical to that used in the previous model.

Both linear regression models above were also fitted without any temperature predictors. Akaike information criterion (AIC) values were calculated for model selection, and the Shapiro-Wilk test was used to confirm that the residuals of each model were normally distributed.
