# Supplementary material for: Size, not temperature, drives cyclopoid copepod predation of invasive mosquito larvae
Source: PLoS One. 2021 Feb 2;16(2):e0246178. doi: 10.1371/journal.pone.0246178 (PMC7853444; doi:10.1371/journal.pone.0246178)
Supplement: S4 File — (PDF) [file pone.0246178.s014.pdf]

S4 File: Copepod body sizes by species and experimental design

The ten gravid female copepods of each species that were measured as a validation set for the size of adult females included in the functional response experiments ranged from 1.2 to 1.7 mm for *M. albidus,* and from 1.4 to 2.3 mm for *M. viridis*. The non-gravid copepods used as predators in these experiments ranged from 1.2 to 1.9 mm (n = 81) for *M. albidus*, and from 1.4 to 2.5 mm (n = 80) for *M. viridis*. The eighty-one *M. albidus* copepods included in the functional response curve experiments ranged from 0.09 to 0.32 mg in body mass (median = 0.20 mg), and the eighty *M. viridis* copepods included in the functional response curve experiments ranged from 0.14 to 0.67 mg in body mass (median = 0.32 mg). Results of the Shapiro-Wilk test showed that the distribution of body masses included in the functional response experiments was significantly different from a normal distribution for both *M. albidus* (p-value = 0.0019) and *M. viridis* (p-value = 0.0001). The results of a Wilcoxon rank sum test showed that *M. viridis* used in the functional response experiments were generally larger than *M. albidus* used in the same experiments (p-value < 0.0001, S4 Fig).

Twenty-four *M. albidus* copepods were used as predators in the predation efficiency experiments, ranging in body mass from 0.14 to 0.36 mg (mean = 0.24 mg, standard deviation = 0.06 mg), and 23 *M. viridis* were used, ranging in body mass from 0.23 to 0.67 mg (mean = 0.48 mg, standard deviation = 0.10 mg). Results of the Shapiro-Wilk test showed that the distribution of body masses included in the predation efficiency experiments was not significantly different from a normal distribution for both *M. albidus* (p-value = 0.0665) and *M. viridis* (p-value = 0.2128). The results of a Welch two sample t-test for samples of unequal variance showed that *M. viridis* used in the predation efficiency experiments were significantly larger than *M. albidus* used in the same experiments (p-value < 0.0001, S4 Fig).

The Wilcoxon rank sum test was used to identify differences in copepod body mass by experimental design, controlling for species. Copepods used in the predation efficiency experiment were generally larger than those used in the functional response experiment for both *M. albidus* (p-value = 0.0008) and *M. viridis* (p-value = 0.0003).
